# Differentially Private Mobile Crowd Sensing Considering Sensing Errors

**DOI:** 10.3390/s20102785

**Published:** 2020-05-14

**Authors:** Yuichi Sei, Akihiko Ohsuga

**Affiliations:** 1 Department of Informatics, Graduate School of Informatics and Engineering, University of Electro-Communications, Chofu, Tokyo 182-8585, Japan; ohsuga@uec.ac.jp; 2 JST, PRESTO, Kawaguchi, Saitama 332-0012, Japan

**Keywords:** crowdsensing, differential privacy, data mining, sensing errors

## Abstract

An increasingly popular class of software known as participatory sensing, or mobile crowdsensing, is a means of collecting people’s surrounding information via mobile sensing devices. To avoid potential undesired side effects of this data analysis method, such as privacy violations, considerable research has been conducted over the last decade to develop participatory sensing that looks to preserve privacy while analyzing participants’ surrounding information. To protect privacy, each participant perturbs the sensed data in his or her device, then the perturbed data is reported to the data collector. The data collector estimates the true data distribution from the reported data. As long as the data contains no sensing errors, current methods can accurately evaluate the data distribution. However, there has so far been little analysis of data that contains sensing errors. A more precise analysis that maintains privacy levels can only be achieved when a variety of sensing errors are considered.

## 1. Introduction

**Figure 1:**
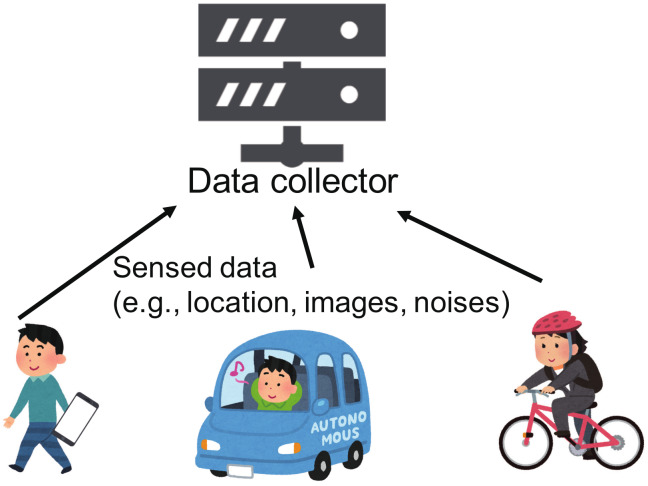
Participatory sensing.

Today’s smartphones are powerful minicomputers that contain an impressive array of sensing components such as cameras or accelerometers, with the ability to collect and analyze users’ surrounding information [1] (Figure 1). Extensive research shows that as well as through mobile phones, data is collected through different means of transportation, such as trains, cars or bicycles. Such information collection is referred to as participatory sensing or mobile crowdsensing. Many studies have been conducted on participatory sensing. For example, Bridgelall et al. proposed a system that detects anomaly locations of roadways using participatory vehicle sensors [2]. Kozu et al. developed a hazard map of bicycle accidents based on data from accelerometers of participatory smartphones [3].

Although high participation is necessary for participatory sensing to be successful, participants may be discouraged by privacy concerns or having to use extra battery power. As such, it is necessary to develop a participatory sensing method featuring both low battery power requirements and high privacy protection [4].

Several frameworks use geotagged posts of Twitter and/or Instagram [5,6]. Although Twitter and Instagram users disclose their locations intentionally, a privacy mechanism could motivate the users to share more geotagged posts.

Several privacy-preserving techniques have been proposed for participatory sensing, such as in References [7,8]. By perturbing data based on ϵ-differential privacy [9,10] privacy leakage can be controlled. Differential privacy has been used in many studies, such as References [11,12,13], as it is one of the strongest privacy metrics [14].

It is problematic, however, that although most collected data contain sensing errors, these seem to have been overlooked in the majority of existing studies. Therefore, the methods used in existing studies reconstruct not the true values but the sensing values with sensing errors (see Table 1). As such, the accuracy of the analysis based on current methods is compromised.

In this paper, we propose an architecture of privacy-preserving participatory sensing considering sensing errors. The proposed architecture consists of two parts. One is the anonymization technique on each participant’s side (perturbing data with sensing errors [PDE]). Each device perturbs its sensed data and then reports the perturbed data to the data collector. Because perturbed data is reported to the data collector, the data collector cannot know the true data distribution. Therefore, the proposed architecture also provides an estimation technique, which estimates the true data distribution based on the reported data, on the data collector’s side (estimating true distribution considering sensing errors [ETE]).

## 2. Models

We define our proposed model. This model is the same as that used in an existing study [8] except for sensing errors.

### 2.1. Application Model

**Figure 2:**
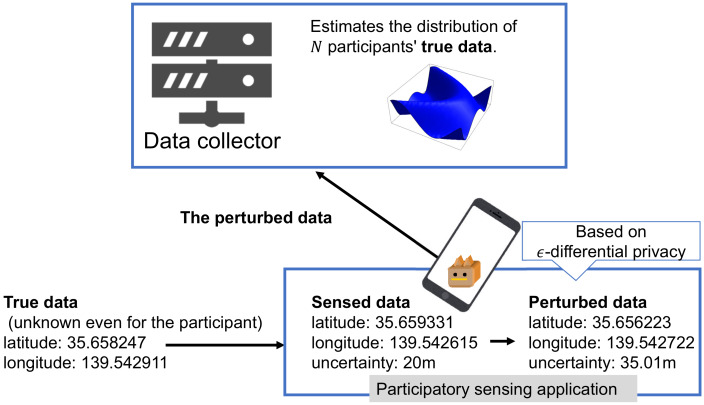
Overview of the proposed architecture.

Sensed data on participants’ surrounding environment that features some sensing errors, such as their location or the radiation level, is collected on mobile phones and sent to the data collector. It is then assumed that the data collector’s analysis results in an accurate data distribution (see Figure 2).

Many factors are worth considering when developing mobile crowdsensing applications, such as radiation levels, urban planning, class of vehicle (for example, whether it is a flatbed truck, taxi or ambulance), and anonymous driver monitoring, as well as more general information such as the participant’s city of residence, surrounding noise levels or personal data such as age and gender [15,16].

There are several stages to the mobile crowdsensing application process. First, the crowdsensing application ID is determined by the data collector so that a selection of crowdsensing applications can be used simultaneously and still be easily distinguishable. Following from this, the data collector must source participants who own an electronic mobile device such as a GPS device or smartphone. Once a participant has volunteered to collaborate with the crowdsensing application, then PVE, the suggested anonymization algorithm, is applied. The final stage is for the data collector to analyze the mass of data using the ETE.

Because several studies suggest that measurement errors follow a normal distribution [17], it is used in this paper as an error model. Standard deviation is defined by the parameter σ, which typifies normal distribution. It is widely recognized that true sensing data falls within the normal distribution pattern [18,19,20]. Indeed, a study of 29,000 items of GPS data collected by Devon et al. [21] and real-time gesture recognition achieved by the pose tracking accuracy of the Microsoft Kinect 2 reported by Wang et al. [22] both follow normal distribution patterns.

It can thus be predicted that error probability also, for the most part, emulates a normal distribution pattern. The accuracy of a sensor is normally depicted on a data sheet shown by sensor vendors. For example, a standard deviation of a normal distribution is shown on the data sheet. If an average error is shown, we can obtain the standard deviation of the normal distribution.

Jiang et al. proposed a fault diagnosis system that took into account a measurement error problem [23]. They assumed that the measurement error usually follows a normal distribution. Wang et al. proposed a measurement system for the rotational angle of the wheel [24]. They considered sensing error analysis to be a very important problem. Location errors of an accelerometer were set to follow normal distributions in their experiments.

MPU-6000 IMU is a low-cost navigation system for ground vehicles. Gonzalez et al. [25] collected real sensing data from MPU-6000 IMU and concluded that sensing errors of accelerometers of ZMPU-6000 follow normal distributions, and sensing errors of gyroscopes of ZMPU-6000 can be modeled as pseudo normal distribution processes, although the errors do not follow a perfect, normal distribution. They also collected real sensing data of Ekinox IMU. They showed that sensing errors of accelerometers and gyroscopes followed normal distributions.

Nguyen et al. discussed how sensing location errors affects mobile, robotic, wireless sensor networks [26]. The sensing location errors were modeled to follow normal distributions in their proposed algorithm. They showed that their algorithm realized a high performance using real data sets containing sensing errors.

Similarly, a machine learning technique featuring deep neural networks has been adopted by sensing systems. Several studies based on deep neural networks reported that prediction errors followed a normal distribution [27,28,29]. If several training samples can be amassed, a data collector can analyze the standard deviation of the error distribution.

Although not all sensing errors follow normal distributions, many sensing errors are considered to follow normal distributions, as described above. Our proposed method targets the situation in which sensing errors can be considered to follow normal distributions.

### 2.2. Motivating Example

Assume that the data collector wants to analyze the noise level in each location to tackle a plane noise problem. To increase the number of participants, the data collector wants to mitigate the privacy issues of the participants. In this case, each participant can perturb her/his location information, and then each participant reports the perturbed location information. Because the reported location information is perturbed by each participant, the data collector should reconstruct the true information. However, because existing studies did not consider the sensing errors of the true location information, the accuracy of the reconstructed location information with the data collector will decrease.

In this paper, we aim to increase the accuracy of the reconstructed information with the data collector by modeling the sensing errors at the participants’ side.

### 2.3. Privacy Metric

Extensive research completed within publications in the data-mining field [30,31,32] reveals that differential privacy [33] is among the most powerful privacy measures available. The following context can be considered: an honest data holder with a database containing participants’ true information is paired with a malicious data analyst desiring access to that database. Whenever the database is accessed by the analyst, noise is added to the query response based on a privacy mechanism A. The differential privacy can be understood in the following manner, with ϵ as a positive real number:

**Definition** **1** (*ϵ*-differential privacy)**.** *Databases D and $D^{\prime }$ are neighboring databases, if they differ only in at most one record. A privacy mechanism A satisfies ϵ-differential privacy if and only if for any output Y, the following equation holds for all databases of D and $D^{\prime }$:*

$$P\left(A\left(D\right)\in Y\right)\leq e^{\epsilon }P\left(A\left(D^{\prime }\right)\in Y\right),$$

*where Y⊆Range(A).*

This method can be used for privacy-preserving participatory sensing [34]:

**Definition** **2** (local privacy)**.** *Databases x and $x^{\prime }$ are neighboring databases with size = 1. A privacy mechanism A satisfies ϵ-differential privacy if and only if for any output y, the following equation holds for all databases of x and $x^{\prime }$:*

(1) $$P\left(A\left(x\right)=y\right)\leq e^{\epsilon }P\left(A\left(x^{\prime }\right)=y\right).$$

### 2.4. Utility Metric

Data analysis can be achieved through the data collector producing data distribution, which is expressed by a (multidimensional) histogram or a cross-tabulation. To measure the difference between the original data generated distribution, information known to neither the participant nor the data collector, and the reported data generated distribution analyzed by the data collector, the utility metric Mean Squared Error (MSE) is employed.

Let *N* denote the number of participants, and let $H_{1},H_{2},\ldots ,H_{b_{n}}$ denote each bin of a histogram of sensed data. Here, $b_{n}$ represents the number of bins. Let ${§}_{j}$ denote the number of participants whose true data is categorized to $H_{j}$, and let ${‡}_{i}$ denote the number of participants categorized to $H_{i}$ in the estimated histogram at the data collector.

**Definition** **3** (MSE)**.** *We use the MSE between ${‡}_{j}$ and ${§}_{j}$ to quantify the utility for the estimated histogram:*

(2) $$MSE=\frac{1}{b_{n}}\sum_{j=1}^{b_{n}}{\left({§}_{j}-{‡}_{j}\right)}^{2}.$$

### 2.5. Problem

The objective is to ensure the ϵ-differential privacy is achieved, each sensed value is anonymized, and a (multidimensional) histogram is created, while the MSE remains minimized to retain superior quality. This is outlined below:

**Problem** **1.** *Given a set of participants U (the size of U is N), their sensed data $x_{i}$(i=1,…,N), and a privacy parameter ϵ, find anonymized data $y_{i}$ satisfying ϵ-differential privacy for all i. Moreover, given the anonymized data $y_{i}$, find estimated data distribution ${‡}_{i}$$\left(i=1,\ldots ,b_{n}\right)$ that minimizes the MSE.*

## 3. Related Work

### 3.1. Privacy-Preserving Mobile Crowdsensing

Several privacy-preserving systems including [35,36,37] that are based on encryption, known as encryption schemes, can be established for this context. They assume that the data collector might be a malicious entity but that the participant fraction conspiring together with the data collector, can be no higher than the predefined value γ. Honest participants’ private data could be leaked if the data collector connives with over γ% of participants. It must also be highlighted that, as demonstrated in Section 2.1, it is quite simple for data collectors to create smartphone emulators to freely connive within mobile crowdsensing situations.

One increasingly trusted system that safeguards participants’ data regardless of whether data collector and N−1 of *N* participants are conspiring together is randomized response [38]. Here, a sensed value represents a predefined category that is then substituted with a certain probability category before the data collector receives it. In this way, participants’ privacy is to some extent ensured as the true data with probability *p* and the perturbed data with probability 1−p are sent to the server. Although the data collection server cannot obtain reliable information about each participant’s data, by collecting information from many participants and conducting a statistical analysis, it is possible for the data collection server to estimate the true data distribution with some degree of accuracy.

Several methods that extend randomized response have been proposed, such as [7,8,39]. In a method called S2Mb (single to randomized multiple dummies with bayes) [8], each participant selects and reports several category IDs to the data collector. By adjusting the probability of selecting the original category ID and the number of selected category IDs, S2Mb can achieve higher accuracy while maintaining the privacy protection level. S2Mb outperformed other privacy-preserving methods [8].

Task allocation is one of the main issues of mobile crowdsensing. Yang et al. proposed a privacy-preserving framework that can allocate tasks to each participant [40]. They assume that the data collector is a trusted entity, and the data collector has a signed agreement with participants. On the contrary, the data collector in our proposed method does not need such an agreement.

The protecting location privacy (PLP) framework was proposed by Ma et al. [41]. Each participant specifies her/his privacy location in advance, and the participant sends all sensing data with location information as they are, except for the data sensed in the specified privacy location. Because several data are sent to the data collector without any modification, PLP does not satisfy differential privacy. PLP uses another privacy metric named δ-privacy, and satisfying differential privacy is out of the scope of the PLP framework.

In mobile crowdsensing, there is a tradeoff between participants’ privacy and data utility. Gao et al. proposed a game model that addressed this contradictory issue [42]. Their method helps to determine the value of the privacy budget ϵ of differential privacy. As noted in their paper, their method does not care about how to add noises to the sensing data or how to conduct statistical analysis. Our proposed perturbing data with sensing errors (PDE) and estimating true distribution considering sensing errors (ETE) can be used for adding noise and conducting statistical analysis, respectively.

Huai et al. proposed a privacy-preserving aggregation framework [43]. Their method can realize high data utility while preserving privacy. They assume that the data collector might not be a trusted entity. However, if many participants collude with the data collector, an honest participant’s privacy will be leaked to the data collector. Because it is difficult for the honest participant to know how many other honest participants there are, the honest participant still might have privacy concerns.

Huang et al. proposed a privacy-preserving incentive mechanism for mobile crowdsensing [44]. Their target application is a noise monitoring system that collects noise levels and corresponding location information. Because the noise level is related to the location, an attacker could estimate each participant’s location using a location inference attack. Although they did not consider sensing errors, Huang et al.’s proposed mechanism satisfies differential privacy and prevents location inference attacks.

Nonetheless, sensing errors are not taken into account in the methods outlined.

### 3.2. Privacy Metrics

There are many privacy metrics other than differential privacy. For example, *k*-anonymity was originally proposed as a privacy model when publishing medical data [45], and it is used today in many studies [46,47]. *k*-anonymity ensures that there are *k* or more records that have the same quasi-identifier values so that *k*-anonymity can protect against “identity disclosure”. For example, a method wherein a database that originally recorded ages in 1-year increments is abstracted to 30 s, 40 s, and so forth. Even in the event an attacker knows all the quasi-identifier information about a given user, because there are *k* or more records corresponding to that user, they cannot tell beyond a 1/k level of confidence which record belongs to the corresponding user. There are also *k*-anonymity related privacy metrics such as *l*-diversity [48] and *t*-closeness [49]. These privacy metrics are also important; however, applying our proposed model to privacy metrics other than differential privacy is out of the scope of this paper and considered for future work.

### 3.3. Incentive Mechanism and Trustworthiness for Mobile Crowdsensing

An incentive mechanism is a very important issue for mobile crowdsensing. If the incentive mechanism works well, it is expected that the crowdsensing system can gather many participants even if the privacy levels are relatively low. On the other hand, if there are no good incentive mechanisms, the privacy levels should be higher to recruit many participants.

Suliman et al. proposed an incentive-compatible mechanism for group recruitment [50]. They considered the greediness of participants of in-group recruitment, and the proposed mechanism can increase the quality of the collected information by selecting participants who are expected to give high-quality data at a low cost.

A reverse auction mechanism also can be used for recruiting participants. The participants bid their expected rewards, and the crowdsensing manager selects good participants. In general, the winning probability is not known to the participants. Modified reverse auction (MRA) mechanisms proposed by Saadatmand et al. provide the estimated winning probability to participants [51].

The participants can modify their bidding price to increase their probability of winning. Wu et al. proposed a modified Thompson sampling worker selection (MTS-WS) mechanism, which uses reinforcement learning to estimate each participant’s data quality [52].

The prevention techniques against false data injection attacks are also important for the success of mobile crowdsensing. We can use these techniques, such as in References [53,54,55], to select reliable participants who contribute to maximizing the quality of mobile crowdsensing.

Zhang et al. proposed a privacy-preserving crowdsensing framework using an auction mechanism [56]. They assumed that the data collector is a trusted entity, and each participant sends her/his sensitive data to the data collector as-is. Therefore, the privacy information of participants is known to the data collector. On the contrary, we assume that the data collection server might not be a trusted entity. Each participant’s original data need not be sent to any other entities in our proposed method.

There are several important mobile crowdsensing survey articles. Capponi et al. analyzed mobile crowdsensing studies and outlined future research directions [57]. Liu et al. [58] focused on privacy and security, resource optimization, and incentive mechanisms. They argued that ensuring privacy and trustworthiness is important.

Pouryazdan et al. [59] proposed three new metrics to quantify the performance of mobile crowdsensing: platform utility, user utility, and false payments. Using these metrics, they showed that data trustworthiness and data utility could be improved by collaborative reputation scores, which are calculated based on statistical reputation scores and vote-based reputation scores.

Pouryazdan et al. [60] proposed a gamification incentive mechanism. They formulated a game theory approach and showed that their mechanism could improve data trustworthiness greatly. Moreover, the proposed mechanism could prevent the data collector from paying rewards to malicious participants.

Xiao et al. formulated the interactions between the data collector and the participants as a Stackelberg game [61]. Because the sensing accuracy determined the reward, each participant was motivated to sense highly accurate data. Deep Q-Network, a reinforcement learning algorithm with deep neural networks, was used to determine the optimal reward.

Privacy-preserving mechanisms, including our proposed method, could be combined with such incentive mechanisms to increase participants while maintaining a low cost.

Domínguez et al. [5] proposed a method that detects unusual events based on geolocated posts on Instagram. The framework uses DBSCAN, a density-based clustering algorithm that executes an outlier detection algorithm to detect unusual events. INRISCO, an incident detection platform for smart cities, was proposed by Igartua et al. [6]. INRISCO uses Twitter and Instagram posts along with the data of vehicular and mobile ad hoc networks. Although Twitter and Instagram users disclose their locations intentionally, privacy-preserving mechanisms and incentive mechanisms could motivate the users to share more geotagged posts. As a result, the ability to detect unusual events can be improved.

## 4. Method

### 4.1. Overview

We assume that sensing errors follow a probability distribution such as a normal distribution, as described in Section 2.1.

Here, there are two scenarios. In the first scenario, the standard deviation of the sensing error is not considered private information. Because the standard deviation itself does not have any sensitive meaning, this scenario is reasonable. In the second scenario, we consider the standard deviation of the sensing error to also be private information. For example, if the standard deviation is correlated with the sensing value, then the second scenario is preferred. Our proposed architecture can address both scenarios.

A differential private value can be obtained by adding Laplace noise to a target value [9]. Each participant adds a Laplace noise to the sensed value; then, the noised value is reported to the data collector. The data collector estimates the data distribution (see Figure 2) from all of the reported values. If only one person participates in the participatory sensing, then the data collector concludes that the reported value is most likely to be the real value. However, if there are many participants, the data collector can estimate a more accurate data distribution through the statistical analysis proposed in this paper.

Our main notations are summarized in Table 2.

### 4.2. PDE for Participants

In this section, we propose an anonymization technique at each participant’s side: perturbing data with sensing errors (PDE).

The data collector determines the minimum and maximum values of the sensed data for which to use differential privacy. For example, the data collector can determine whether the participant’s noise volume is from 0 to 120 dB. If the sensed value is out of this range, the value is considered to be 0 (if the sensed value is less than 0) or 120 (if the sensed value is greater than 120) on the participant’s device. Let $minv_{org}$ and $maxv_{org}$ represent the minimum and maximum values of the sensed values.

The value range of perturbed data is infinity because a Laplace noise is added to the sensed data. To avoid decreasing the accuracy of an estimated histogram, the data collector also determines the minimum and maximum values of the reported data with which to create a histogram. Let $minv_{rep}$ and $maxv_{rep}$ represent these values.

If the data collector considers the standard deviation of the sensing error to also be private information, then the data collector will determine the minimum and maximum values of the standard deviation. Let $min\sigma_{org}$ and $max\sigma_{org}$ represent these values.

The Laplace mechanism [9] can be used, which adds noise based on the Laplace distribution. The theorem of the Laplace mechanism for data collection is introduced.

**Theorem** **1** (Laplace Mechanism)**.** *A privacy mechanism A realizes ϵ-differential privacy if A adds the Laplace noise Lap(Δ/ϵ), where *Δ* is the range of the target attribute’s possible values, and Lap(b) returns independent Laplace random variables with the scale parameter b.*

If the standard deviation is considered private information, then a Laplace noise is added to the standard deviation as well as to the sensing data.

In the second scenario, in which the standard deviation σ of the sensing error is considered private information, a Laplace noise is added to not only the sensed value *x* but also the value of σ. If two elements are protected by ϵ-differential privacy, we should divide the privacy budget ϵ into two elements [34].

Algorithm 1 shows the PDE algorithm.
**Algorithm 1** Anonymization Algorithm.**Input:**$minv_{org}$, $maxv_{org}$, $minv_{rep}$, $maxv_{rep}$, $min\sigma_{org}$, $max\sigma_{org}$, ϵ.**Output:** Report value *v* and standard deviation σ of sensing error1:Obtain sensed value *v* and standard deviation σ of sensing error2:**if** the standard deviation is considered as private information **then**3:ϵ←ϵ/24:**end if**5:$v\leftarrow \min(\max\left(minv_{org},v\right),maxv_{org})$  /* If *v* is smaller than $minv_{org}$ (or larger than $maxv_{org}$), *v* is set to $minv_{org}$ (or $maxv_{org}$).*/6:$v\leftarrow v+Lap(\left(maxv_{org}-minv_{org}\right)/\epsilon )$  /* The global sensitivity is $maxv_{org}-minv_{org}$.*/7:$v\leftarrow \min(\max\left(minv_{rep},v\right),maxv_{rep})$  /* If *v* is smaller than $minv_{rep}$ (or larger than $maxv_{rep}$), *v* is set to $minv_{rep}$ (or $maxv_{rep}$).*/8:**if** the standard deviation is considered as private information **then**9:$\;\sigma \leftarrow \min(\max\left(min\sigma_{org},\sigma \right),max\sigma_{org})$  /* If σ is smaller than $min\sigma_{org}$ (or larger than $max\sigma_{org}$), σ is set to $min\sigma_{org}$ (or $max\sigma_{org}$).*/10:$\;\sigma \leftarrow \sigma +Lap(\left(max\sigma_{org}-min\sigma_{org}\right)/\epsilon )$  /* The global sensitivity is $max\sigma_{org}-min\sigma_{org}$.*/11:**end if**12:Report *v* and σ.

First, a sensing device for each participant measures target data. The device obtains the sensed value *v* and the standard deviation σ (Line 1). If the standard deviation is considered to be private information, the privacy budget ϵ is divided by two (Line 2).

If *v* is smaller than $minv_{org}$, *v* is set to $minv_{org}$, and if *v* is larger than $maxv_{org}$, *v* is set to $maxv_{org}$ (Line 5). Then, PDE adds a Laplace noise to *v* to satisfy ϵ-differential privacy (Line 6). Here, the global sensitivity is $maxv_{org}-minv_{org}$.

Finally, if the value of *v* with Laplace noise is smaller than $minv_{rep}$ (or larger than $maxv_{rep}$), *v* is set to $minv_{rep}$ (or $maxv_{rep}$) (Line 7). If the standard deviation σ is considered to be private information, PDE adds a Laplace noise to σ (Line 10).

**Theorem** **2.** *The proposed PDE realizes ϵ-differential privacy.*

**Proof.**  The global sensitivity $\Delta_{v}$ of a sensing value and the global sensitivity of the standard deviation of a sensing error $\Delta_{\sigma }$ are $\left(maxv_{org}-minv_{min}\right)$ and $\left(max\sigma_{org}-min\sigma_{org}\right)$, respectively. According to Theorem 1, when a Laplace noise with scale $\Delta_{v}/\epsilon$ is added to the sensing value, we can achieve ϵ-differential privacy with regard to the sensing value. Similarly, when a Laplace noise with scale $\Delta_{\sigma }/\epsilon$ is added to the standard deviation of the sensing error, we can achieve ϵ-differential privacy with regard to the standard deviation.

When we consider the standard deviation to be private information, we should achieve ϵ-differential privacy for the combination of the sensing value and the standard deviation. In this case, PDE achieves ϵ/2-differential privacy with regard to the sensing value and the standard deviation, respectively. Therefore, according to Reference [34], PDE achieves ϵ-differential privacy in total. □

### 4.3. ETE for Estimation

In this section, we propose an estimation technique that estimates the true data distribution based on the reported data, at the data-collector side: estimating true distribution considering sensing errors (ETE).

The data collector estimates the true data’s distribution, which is represented by a (multi-dimensional) histogram, from the reported data. Each true data point of each participant might be unknown to the participant.

Let F(y;x,θ) be the probability density function with regard to *y*, which represents the reported sensing value, where *x* represents the true value and θ represents the set of parameters comprising the sensing error and a Laplace noise.

Let $x_{i}$ and $y_{i}$ represent the true sensing value and the reported sensing value of participant *i*, respectively. The value $y_{i}$ contains a sensing error following a normal distribution and a Laplace noise to satisfy ϵ-differential privacy. That is when the true value is $x_{i}$, the probability density with which the reported value becomes $y_{i}$ is $F(y_{i};x_{i},\theta )$. Let *X* and *Y* represent $\left\{x_{1},\ldots ,x_{N}\right\}$ and $\left\{y_{1},\ldots ,y_{N}\right\}$, respectively. Based on F(y;x,θ), by using Bayes’ technique, we can estimate the distribution of *X* from *Y*.

Let *w* be the width of each bin of the histogram. The value of *w* is calculated by

(3) $$w=\frac{maxv_{rep}-minv_{rep}}{b_{n}},$$

where $b_{n}$ represents the number of bins of an estimated histogram, as determined by the data collector.

The function F(y;x,θ) is a probability density function, and *y* is a continuous random variable. The number of samples of *y* is a finite set in a real situation; therefore, we approximate the probability density function as a probability mass function. The domain of *y* is defined as

(4) $$V=(minv_{rep}+w/2,minv_{rep}+2w/2,minv_{rep}+3w/2,\ldots ,minv_{rep}+b_{n}*w/2).$$

Let P be the $b_{n}\times b_{n}$ matrix and P(i,j) represent the value of P in the *i*th row and *j*th column. P(i,j) represents the probability that the reported value is categorized into *j*th bin when the true value is categorized into *i*th bin.

Let ${†}_{i}$ be the number of participants whose reported values are categorized into the *i*’th bin, and let ${‡}_{i}$ be the estimated number of participants whose true values are categorized into the *i*’th bin. Let **†** and **‡** be the sets $\left\{{†}_{1},\ldots ,{†}_{b_{n}}\right\}$ and $\left\{{‡}_{1},\ldots ,{‡}_{b_{n}}\right\}$, respectively.

Based on the iterative Bayes’ technique [62], we have

(5) $${‡}_{i}\leftarrow \sum_{j=1}^{b_{n}}{†}_{j}\frac{P\left(i,j\right){‡}_{i}}{\sum_{k=1}^{b_{n}}P\left(k,j\right){‡}_{k}}.$$

Equation (5) is repeated a sufficient number of times.

Several values of the estimated data distribution might be negative. Therefore, the data distribution should be adjusted so that all values are greater than or equal to zero. The values are perturbed based on the probability simplex algorithm [63]. Moreover, because the data collector determines the value range for sensing in advance, values that are out of range should be zero. Note that to use differential privacy as a privacy metric, we must determine the value range in advance if we use any other methods that can satisfy differential privacy. Therefore, in each iteration, for

(6) $$i\leq \left\lceil \frac{minv_{org}-minv_{rep}}{w}\right\rceil -1,$$

and

(7) $$i\geq \left\lceil \frac{maxv_{org}-minv_{rep}}{w}\right\rceil -1,$$

we set

(8) $${‡}_{i}\leftarrow 0,$$

because the true values are within $minv_{org}$ and $maxv_{org}$.

Now, we describe how to obtain P. Each value of P(i,j) is calculated by the following equation for all values of *i*;

(9) $$\left\{\begin{array}{c} P\left(i,1\right)=\int_{-\infty }^{minv_{rep}+w}F\left(y;minv_{rep}+\left(i-1\right)*w+w/2,\theta \right)dy\\ P\left(i,j\right)=\int_{minv_{rep}+\left(j-1\right)*w}^{minv_{rep}+j*w}F\left(y;minv_{rep}+\left(i-1\right)*w+w/2,\theta \right)dy\;\;\mathrm{for}\;\;j=2,\ldots ,b_{n}-1\\ P\left(i,b_{n}\right)=\int_{minv_{rep}+\left(b_{n}-1\right)*w}^{\infty }F\left(y;minv_{rep}+\left(i-1\right)*w+w/2,\theta \right)dy. \end{array}\right.$$

The function F(y;x,θ) differs for the two scenarios. First, we consider the scenario in which the standard deviation of an error distribution is not private information. That is, a Laplace noise is added to the sensed value before the value is reported to the data collector, but each participant reports the standard deviation of the sensing error as it is to the data collector. In this case, the data collector can determine the true standard deviation of the sensing error’s normal distribution. Let $b_{v}$ be a scale factor of a Laplace noise with regard to the sensed value. The value $b_{v}$ is represented by

(10) $$b_{v}=\frac{maxv_{org}-minv_{org}}{\epsilon },$$

and we can consider $\theta =\{\sigma ,b_{v}\}$.

In this case,

(11) $$F\left(y;x,\theta \right)=F\left(y;x,\sigma ,b_{v}\right)=\int_{-\infty }^{\infty }N\left(t;x,\sigma \right)*L\left(y;t,b_{v}\right)dt,$$

where N(t;x,σ) represents the probability density of *t* in a normal distribution with a mean of *x* and a standard deviation of σ, and $L(y;t,b_{v})$ represents the probability density of *y* in a Laplace distribution with a mean of *t* and a scale factor of $b_{v}$.

In the second scenario, where the standard deviation σ of the sensing error is considered to be private information, a Laplace noise is added to not only the sensed value *x*, but also the value σ, as described in Section 4.2.

Let $b_{v}$ and $b_{\sigma }$ be scale factors of a Laplace noise with regard to the sensed value and the standard deviation, respectively. The values $b_{v}$ and $b_{\sigma }$ are represented by

(12) $$b_{v}=\frac{maxv_{org}-minv_{org}}{\epsilon /2},$$

and

(13) $$b_{\sigma }=\frac{max\sigma_{org}-min\sigma_{org}}{\epsilon /2}.$$

In this case, we consider $\theta =\{\sigma ,b_{v},b_{\sigma }\}$, and obtain

(14) $$\begin{array}{cc} \; & F\left(y;x,\theta \right)=F\left(y;x,\sigma ,b_{v},b_{\sigma }\right)\\ \; & =\int_{-\infty }^{\infty }\int_{0}^{\infty }N\left(t;x,u\right)*L\left(y;t,b_{v}\right)*L\left(\sigma ;u,b_{\sigma }\right)dudt/V, \end{array}$$

where

(15) $$V=\int_{-\infty }^{0}L\left(x;\sigma ,b_{\sigma }\right)dx=e^{-\sigma /b_{\sigma }}/2.$$

**Figure 3:**
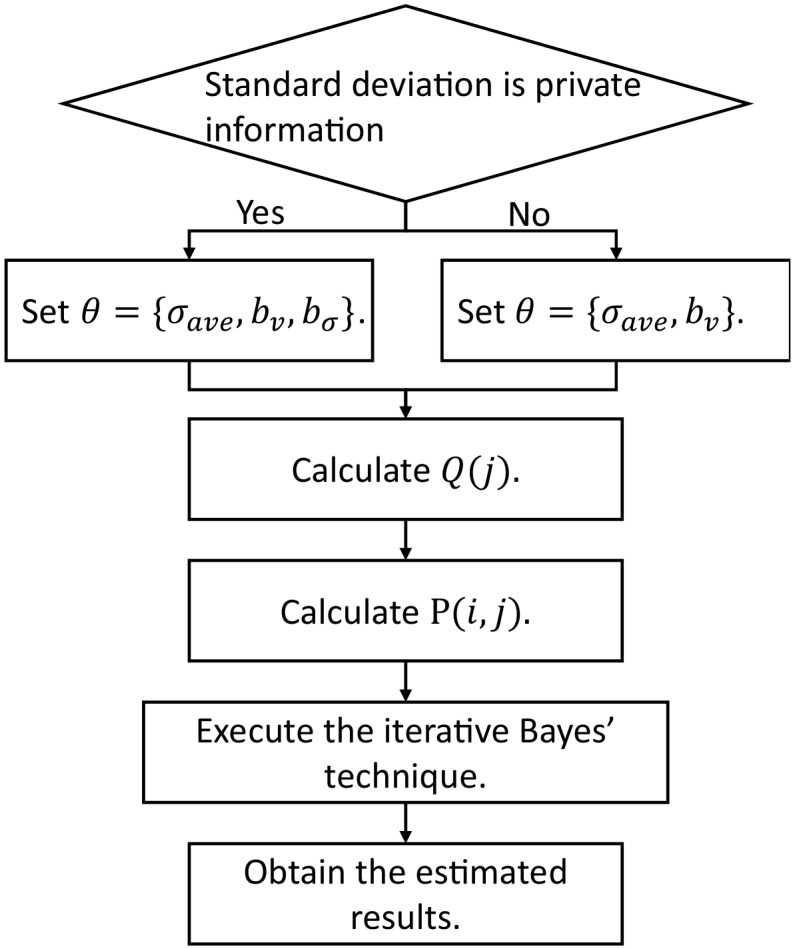
A high-level diagram of the estimation algorithm.

Figure 3 shows a high-level diagram of the estimation algorithm (ETE) and Algorithm 2 shows the details.
**Algorithm 2** Estimation Algorithm.**Input:***Y*, $Y_{\sigma }$, ϵ, $minv_{org}$, $maxv_{org}$, $minv_{rep}$, $maxv_{rep}$, $min\sigma_{rep}$, $max\sigma_{rep}$, $b_{n}$**Output:**‡1:$\sigma_{ave}\leftarrow Average\left(Y_{\sigma }\right)$  /* Consider $\sigma_{ave}$ is the standard deviation of each participant*/2:**if** standard deviation is considered as private information **then**3:$\;b_{v}$ and $b_{\sigma }$ are calculated by Equations (12) and (13), and set $\theta =\{\sigma_{ave},b_{v},b_{\sigma }\}$.4:**else**5:$\;b_{v}$ is calculated by Equation (10), and set $\theta =\{\sigma_{ave},b_{v}\}$.6:**end if**7:$w\leftarrow \left(maxv_{rep}-minv_{rep}\right)/b_{n}$  /*w represents the width of each bin*/8:x← an arbitrary real number9:$left\leftarrow \int_{-\infty }^{x-w/2}F\left(y;x,\theta \right)dy$10:**for**$j=1,\ldots ,b_{n}$**do**11:$\;Q\left(j\right)\leftarrow \int_{x-w/2+(j-1)*w}^{x-w/2+j*w}F\left(y;x,\theta \right)dy$  /*P(1,j)=Q(j) for $j=2,\ldots ,b_{n}-1$. P(1,1)=left+Q(1). $P\left(1,b_{n}\right)=Q\left(b_{n}\right)+right$.*/12:**end for**13:$right\leftarrow 1-left-\sum_{j=1}^{b_{n}}Q\left(j\right)$14:**for**$i=1,\ldots ,b_{n}$**do**15: $P\left(i,1\right)\leftarrow left+Q\left(1\right)-\sum_{j=1}^{i-1}Q\left(j\right)$  /*Note that $\sum_{j=1}^{0}Q\left(j\right)=0$*/16: **for** $j=2,\ldots ,b_{n}-1$ **do**17:  P(i,j)←Q(|i−j|+1)
18: **end for**19:$\;P\left(i,b_{n}\right)\leftarrow \sum_{j=b_{n}-i+1}^{b_{n}}Q\left(j\right)+right$20:**end for**21:Set ${†}_{i}$ for each *i* based on *Y*.22:**for** Repeat sufficient times **do**23: **for** $i=1,\ldots ,b_{n}$ **do**24:  $d_{i}\leftarrow 0$
25:  **for** $j=1,\ldots ,b_{n}$ **do**26:$\;d_{i}\leftarrow d_{i}+P\left(k,j\right)*{‡}_{k}$  /*Calcuation of the denominator of Equation (5)*/27:  **end for**28: **end for**29: **for** $i=1,\ldots ,b_{n}$ **do**30:  **for** $j=1,\ldots ,b_{n}$ **do**31:$\;{‡}_{i}^{\prime }\leftarrow y_{j}*P\left(i,j\right)/d_{j}$32:  **end for**33:$\;{‡}_{i}\leftarrow {‡}_{i}*{‡}_{i}^{\prime }$34: **end for**35: **for** $i=1,\ldots ,b_{n}$ **do**36:  **if** $i\leq \left\lceil \frac{minv_{org}-minv_{rep}}{w}\right\rceil -1\;\;\mathrm{OR}\;\;\left\lceil \frac{maxv_{org}-minv_{rep}}{w}\right\rceil -1\leq i$ **then**37:$\;{‡}_{i}\leftarrow 0$38:  **end if**39: **end for**40:**end for**

Because the values of P(i,j) ($i=1,\ldots ,b_{n}$ and $j=2,\ldots ,b_{n}-1$) are the same when the values |i−j| are the same, we calculate only P(1,j) (represented by Q(j)) and additional values represented by left and right in lines 9–13. Then, we construct P(i,j) in lines 14–20.

**Figure 4:**
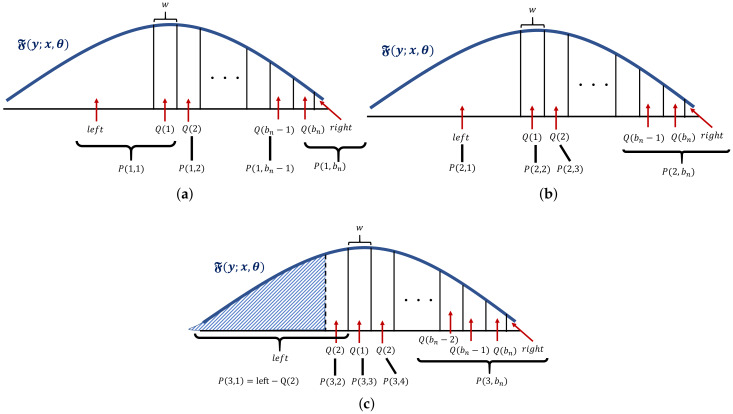
Relationship between Q(j) and P(i,j). (**a**) Relationship between Q(j) and P(1,j). (**b**) Relationship between Q(j) and P(2,j). (**c**) Relationship between Q(j) and P(3,j). P(3,1) represents the shaded area.

Figure 4a represents the relationship between P(1,j) and Q(j). Each value of Q(j) represents the area marked by the corresponding arrow. The curve line represents the F(y;x,θ). The value of *x* in Algorithm 2 can be arbitrary but is set to the middle of the area, represented by Q(1). Because the summation of $left+right+\sum_{j=1}^{b_{n}}Q\left(j\right)$ is equal to one, we obtain the value of right by $1-left-\sum_{j=1}^{b_{n}}Q\left(j\right)$ in Line 13.

Figure 4b,c represent the relationship between P(2,j) and Q(j) and the relationship between P(3,j) and Q(j), respectively. As increases *i*, P(i,1) decreases, and $P(i,b_{n})$ increase.

Lines 23–34 show the iterative Bayes’ technique. Line 25–27 calculates the value of the denominator of Equation (5). Lines 30–32 calculate the fraction of Equation (5). Finally, the summation of Equation (5) is calculated by Line 33.

Lines 35–39 show the process of Equations (6)–(8).

## 5. Evaluation

Our proposed architecture models sensing errors. If we do not consider the sensing errors, then we consider that only a Laplace noise is added to the true data, even if the sensed data differs from the true data in a real situation. To verify the usefulness of considering the sensing errors, we developed a method of considering only the Laplace noise. We refer to this method as the Laplace mechanism. In this section, we compare our proposal with the Laplace mechanism and with S2Mb, which is described in Section 3. The Laplace mechanism, S2Mb, and the proposed method all use iterative Bayes’ technique. We set the iteration times as the best values for each method, for each simulation, within 100,000 iterations.

The source code for the proposed architecture can be obtained from https://uecdisk.cc.uec.ac.jp/index.php/s/WfIyH8hRMhoF01R. This source code consists of the server (data collector) program and the client (participant) program.

Apple’s deployment ensures that ϵ is equal to 1 or 2 per each datum [64], and that the total privacy loss is 16 per day. An Apple differential privacy team set ϵ=2,4,8 for its evaluations [65]. Based on these settings, ϵ is set in the range 1–15 in the experiments.

### 5.1. Evaluation of Synthetic Data

First, we evaluated the MSE using synthetic datasets. We conducted experiments using several distributions to determine how different data distributions would affect the results. We used three distributions: normal, uniform, and peak. In the uniform distribution, all values of ${§}_{i}$ were set to the same value. In the normal distribution, the values of ${§}_{i}$ followed a normal distribution. In the peak distribution, all of the participants had the same true value.

**Figure 5:**
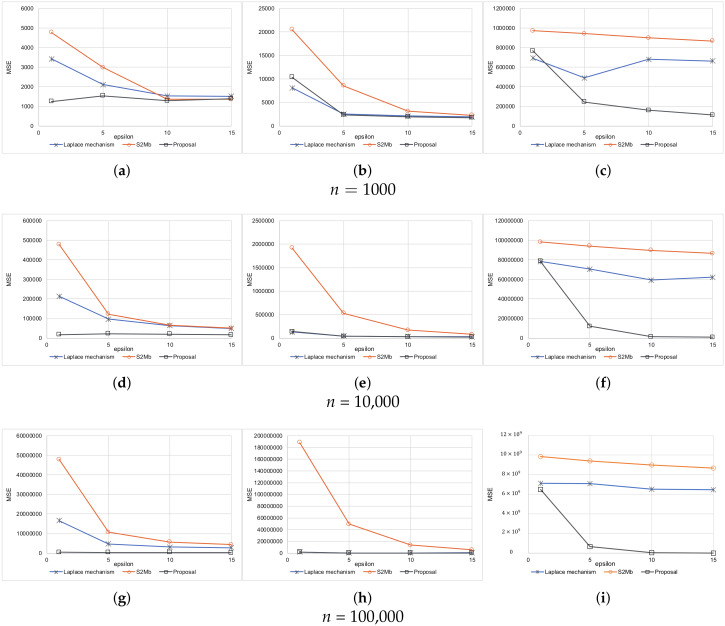
Results of synthetic data (the standard deviation is not private information). (**a**) Uniform. (**b**) Normal. (**c**) Peak. (**d**) Uniform. (**e**) Normal. (**f**) Peak. (**g**) Uniform. (**h**) Normal. (**i**) Peak.

**Figure 6:**
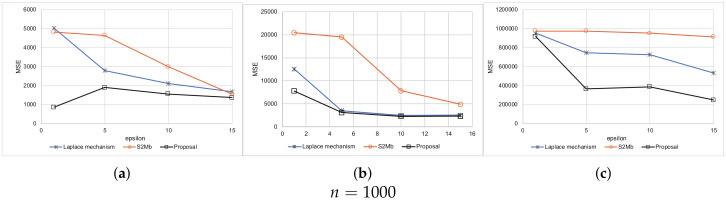

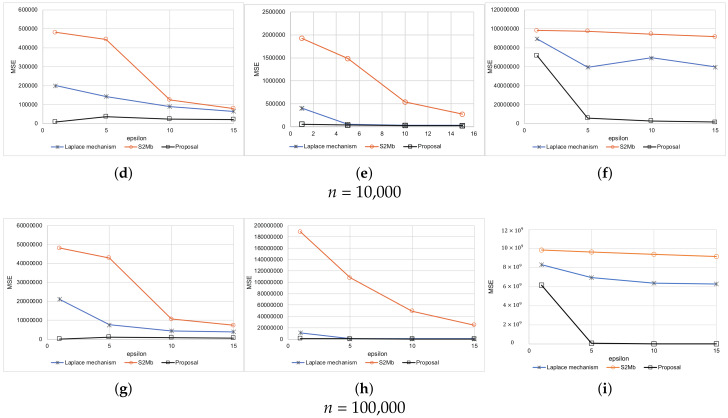
Results of synthetic data (the standard deviation is private information). (**a**) Uniform. (**b**) Normal. (**c**) Peak. (**d**) Uniform. (**e**) Normal. (**f**) Peak. (**g**) Uniform. (**h**) Normal. (**i**) Peak.

Every setting was executed 10 times. The average results are shown in Figure 5 for when the standard deviation of sensing errors is not considered private information. Because the MSEs measure the difference between the true number of people and the estimated number of people within each bin, the MSEs become larger as the number of participants *N* becomes larger. A large value of ϵ means a low privacy-protection level. Therefore, when ϵ is large, the MSEs tend to become small for all methods. Figure 6 represents the experimental results when the standard deviation of the sensing errors is considered private information. Because the standard deviation should be protected in the same way as the sensed values in this situation, the MSEs are larger than those of the results in Figure 5. In all of the settings, the MSEs of our proposed architecture were the smallest among the three methods.

We measured the calculation time at the data collection server’s side. All of the experiments were conducted on a desktop PC with an Intel i7-4770 CPU and 16 GB of RAM. The average calculation time was less than 1 s for the Laplace mechanism and for S2Mb. Our proposed ETE required 14.7 s for each simulation, on average. Although the calculation time of the proposed method is longer than those of the other methods, we believe that the time does not greatly impact the data analysis because gathering participants takes a much longer time (for example, a few days).

### 5.2. Evaluation of Real Data

#### 5.2.1. Location Data

We implemented our proposed PDE as a smartphone application for Android to obtain real sensing data with sensing errors and to verify the algorithm’s feasibility.

Operating systems such as iOS and Android express location by latitude, longitude, and uncertainty (https://developer.apple.com/documentation/corelocation/cllocation [Accessed on 26 March 2020], https://developer.android.com/reference/android/location/Location [Accessed on 26 March 2020]). Uncertainty means a radius of a circle centered at the location’s latitude and longitude, and the true location is inside the circle with 68% probability. In a normal distribution, 68% of the data fall within one standard deviation from the mean.

**Figure 7:**
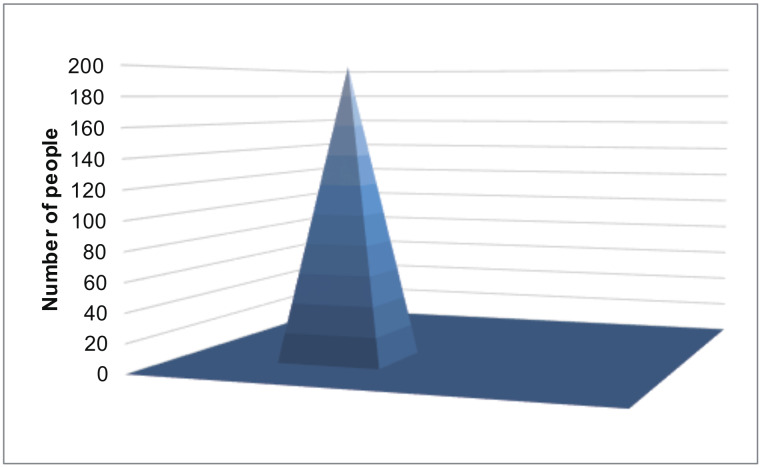
The distribution of the participants’ true locations.

**Figure 8:**
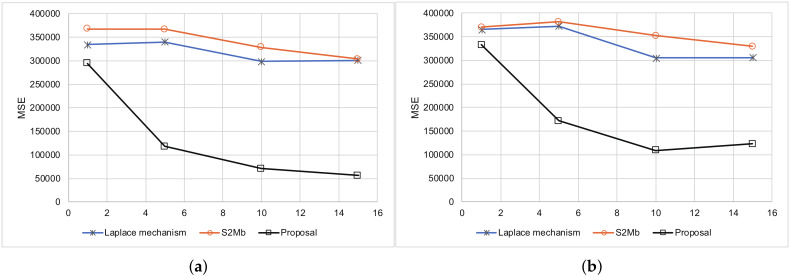
Results summary of the location data. (**a**) Standard deviation is not private information. (**b**) Standard deviation is not private information.

The smartphone was located in the same place and sensed its location along with its uncertainty 200 times. In this experiment, we considered that 200 different people were in the same place. The true distribution of locations is shown in Figure 7. The smartphone reported its differential private location and uncertainty to the data-collection server. We evaluated the MSEs of each method. Figure 8 represents the results. The MSEs of our proposed method were much smaller than those of the other methods.

**Figure 9:**
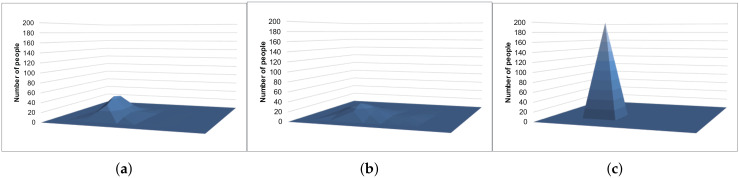
Example results of the location data (the standard deviation is not private information). (**a**) Laplace mechanism. (**b**) S2Mb. (**c**) Proposal.

**Figure 10:**
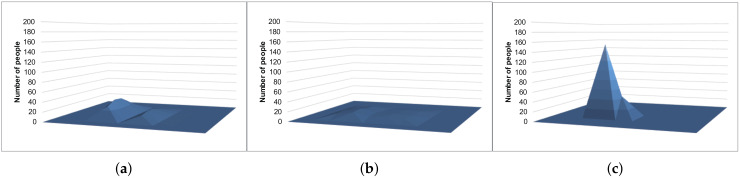
Example results of the location data (the standard deviation is private information). (**a**) Laplace mechanism. (**b**) S2Mb. (**c**) Proposal.

Figure 9 and Figure 10 show the example results of the histograms generated with the Laplace mechanism, S2Mb, and the proposed method. The standard deviation of the sensing errors was considered private information in Figure 10. The histograms of Figure 9c and Figure 10c, which were generated by our proposed architecture, are similar to the true histogram (Figure 7). However, the histograms generated by the Laplace mechanism and S2Mb (Figure 9a,b and Figure 10a,b) are very different from the true histogram.

Furthermore, because some of the participants were concerned about battery consumption [66], we measured the calculation time needed for sensing the GPS and generating differential private data. The smartphone used in this experiment was a SH-M09 with a Snapdragon 845 CPU and 4 GB of RAM. The application was developed with Java. The average time spent for 10 simulations was 100.6 ms. Our PDE is efficient for smartphones, and participants do not need to worry about their smartphones’ battery life.

#### 5.2.2. Deep Neural Network’s Output Data

Crowdsensing might collect an output of a machine learning model, such as deep neural networks (DNNs). For example, each participant’s device can recognize his/her activity from an accelerometer, magnetometer, and gyroscope [67,68] and recognize surrounding people’s age from pictures [69,70]. Surrounding information, such as how many people there are and how old they are, is useful to analyze for a pandemic such as the coronavirus pandemic. For example, age is an important factor for COVID-19 [71,72].

The estimated values from deep neural networks might include estimation errors, and researchers such as [27,28,29] have reported that such estimation errors followed a normal distribution. Several machine-learning models can obtain the probability distribution of a model’s estimated value. For example, the age-estimation model [73] outputs the probability for a person being each age (e.g., the probability of being 1 year old is 0.01%, the probability of being 2 years old is 0.05%, *…*, the probability of being 33 years old is 32.3%, *…*). We developed a deep neural network model that estimates a person’s age from a picture, based on Reference [73].

We assume that it does not make a big difference if the participants report sensing data or an estimated age value. This is because the estimation error of deep neural networks can be considered to follow normal distributions, much as how sensing errors follow normal distributions. We consider that not all estimation errors of deep neural networks follow normal distributions. However, several estimation errors of deep neural networks follow normal distributions, and our proposed method targets such deep neural networks. To confirm that our proposed method can be used for outputs of deep neural networks, this experiment has been conducted.

Table 3 shows the architecture of the deep neural network model we constructed. All of the activation functions of layers are rectified linear units (ReLUs [74]). The loss function was the softmax function. Because our aim is not to increase the accuracy of the deep neural network itself, the accuracy might be increased by tuning architecture or parameters.

**Figure 11:**
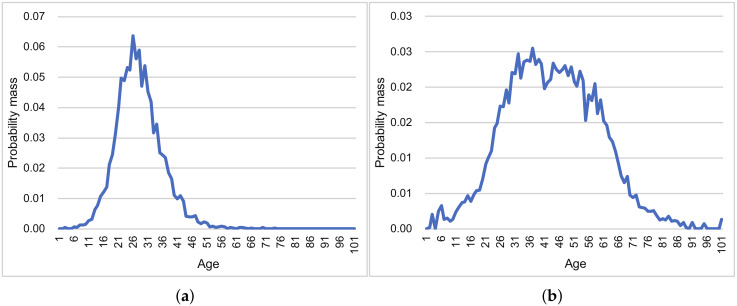
Examples of DNN’s output (probability distribution). (**a**) Example 1. (**b**) Example 2.

We assumed that a crowdsensing application for each smartphone would estimate the surrounding person’s age. Because the model outputs the probability distribution of age, our PDE can calculate the standard deviation of errors at each device. Figure 11 represents the probability distributions of age, which were obtained from the trained deep neural network model. These distributions can be considered as normal distributions.

We used the WIKI dataset, which consists of 22,578 instances (1 GB) (https://data.vision.ee.ethz.ch/cvl/rrothe/imdb-wiki/static/wiki_crop.tar [Accessed on 26 March 2020]). Fifty percent of the dataset was used for our prediction task, that is, we assumed that 11,289 people were the participants. The data collector estimated the true age distribution from the reports. Because each picture in WIKI dataset is labeled true age, we can evaluate the performance of Laplace mechanisms, S2Mb, and the proposal.

**Figure 12:**
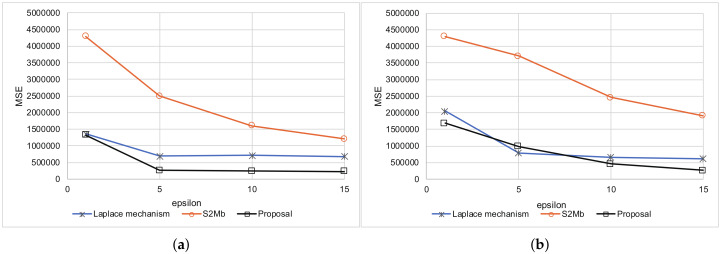
Summary results of age estimation. (**a**) Standard deviation is not private information. (**b**) Standard deviation is private information.

**Figure 13:**
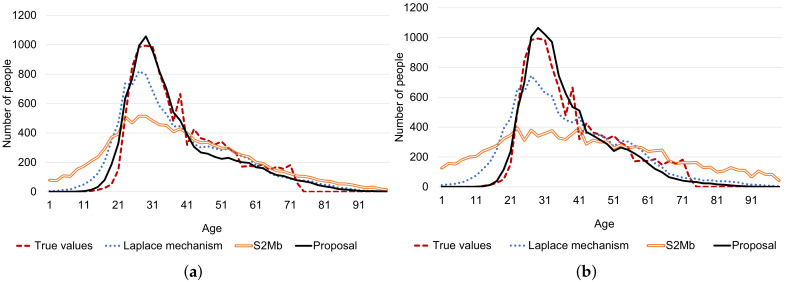
Example results of age estimation. (**a**) Standard deviation is not private information. (**b**) Standard deviation is private information.

Figure 12 summarizes the results of this experiment. In both scenarios, the MSEs of the proposal were smaller than those of the other methods in almost all settings. The true and estimated data distributions are shown in Figure 13. The line of the proposal fits the true values’ line in Figure 13a,b.

## 6. Discussion

In this paper, we assume that the sensing campaigns assign a single sensing task for simple discussion. However, our method can also easily be used for multiple tasks.

**Figure 14:**
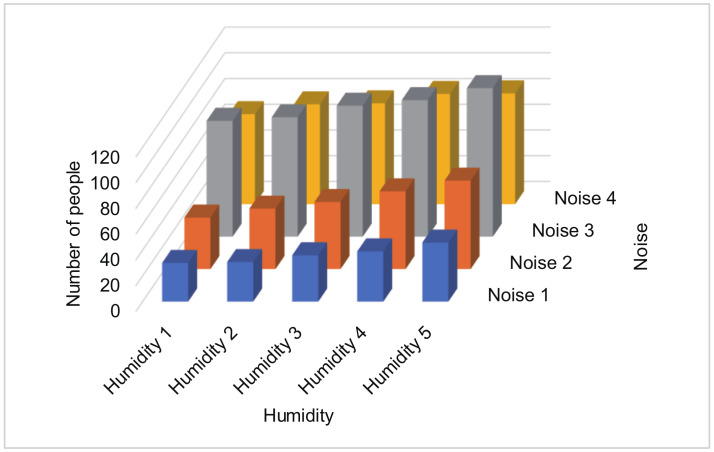
An example of a histogram created by two tasks.

Assume that there are two tasks. For example, the first task is collecting a noise, and the second task is collecting humidity. In this case, we assume that the aim of the data collector is to create a 3D histogram (Figure 14).

Each participant perturbs the two values separately by our proposed PDE method. Then, each participant reports the resulted values and the standard deviations to the data collector. The data collector constructs $P_{1}\left(i_{1},j_{1}\right)$ for the first task (noise sensing) and $P_{2}\left(i_{2},j_{2}\right)$ for the second task (humidity sensing) separately (Lines 1–20 in Algorithm 2). Here, $P_{1}\left(i_{1},j_{1}\right)$ represents the probability that the reported value of the first task is categorized into $j_{1}$th bin when the true value of the first task is categorized into $i_{1}$th bin in the first dimension. In the example in Figure 14, $P_{1}\left(1,2\right)$ represents the probability that the reported value of the noise is “Noise 2” when the true value of the noise is “Noise 1”.

Assume that the number of bins for the first task is $b_{n1}$, and the number of bins for the second task is $b_{n2}$. In the example in Figure 14, $b_{n1}$ = 4 and $b_{n2}$ = 5. The data collector constructs $P_{1,2}\left(\left[i_{1},i_{2}\right],\left[j_{1},j_{2}\right]\right)$ for $i_{1},j_{1}$ = 1, *…*, $b_{n1}$ and $i_{2},j_{2}$ = 1, *…*, $b_{n2}$, which represents that the reported values of the first and second tasks are categorized into $j_{1}$th and $j_{2}$th bins, respectively, while the true values of the first and second tasks are categorized into $i_{1}$th and $i_{2}$th bins, respectively. In the example in Figure 14, $P_{1,2}\left(\left[1,3\right],\left[2,1\right]\right)$ represents the probability that the reported values are “Noise 2” and “Humidity 1,” while the true values are “Noise 1” and “Humidity 3”.

Because each sensed value is perturbed separately, we can calculate $P_{1},2\left(\left[i_{1},i_{2}\right],\left[j_{1},j_{2}\right]\right)$ = $P_{1}\left(i_{1},j_{1}\right)$*$P_{2}\left(i_{2},j_{2}\right)$. Then, the data collector executes the iterative Bayes’ technique using $P_{1},2\left(\left[i_{1},i_{2}\right],\left[j_{1},j_{2}\right]\right)$ (Lines 21–40 in Algorithm 2). Finally, the data collector obtains each estimated number of people in each two-dimensional bin (Figure 14).

## 7. Conclusions and Future Work

Participatory sensing is growing in popularity. Differential privacy can protect a user’s privacy by adding noise to a target value that must be protected. However, in participatory sensing scenarios, the target value contains sensing errors. Because existing studies do not consider the sensing errors, the accuracy of the data analysis decreases when the sensing data contain errors. In this paper, therefore, the proposed architecture can address the noise added to the sensed value. The true data might be unknown to the participants; however, our proposal estimated the participants’ true data distribution with higher accuracy than existing methods by modeling the sensing error.

The proposed architecture consists of two parts. One is the anonymization technique for each participant’s side (PDE). Each device perturbs its sensed data and then reports the perturbed data to the data collector. The proposed architecture also provides an estimation technique, which estimates the true data distribution based on the reported data for the data collector’s side (ETE). We have proved that the PDE satisfies differential privacy. We showed that the accuracy of ETE outperformed existing studies in our experiments. Further, the calculation time of PDE with a normal smartphone was less than 1 s. Therefore, participants do not need to worry about the battery life of their smartphones.

In this paper, we target numerical data with regard to sensing data. Moreover, images can be directly sent to the data collector. In recent years, several methods of protecting images based on differential privacy have been proposed [75]. We will apply our proposal to such data in our future work.

## Figures and Tables

**Table 1:** The difference between the existing methods and our method.

Methods	Output of the Method
Existing methods.	The estimated distribution of sensing data containing errors.
Our method.	The estimated distribution of true data without errors.

**Table 2:** Notations.

*N*	Number of participants.
$x_{i}$	True sensing value of participant *i*.
$y_{i}$	Reported sensing value of participant *i*.
*X*	$\left\{x_{1},\ldots ,x_{N}\right\}$.
*Y*	$\left\{y_{1},\ldots ,y_{N}\right\}$.
$Y_{\sigma }$	Set of standard deviations of the normal distributions of sensing errors of all participants.
$b_{n}$	Number of bins of a histogram.
$maxv_{org}$	Maximum value of a sensing data.
$minv_{org}$	Minimum value of a sensing data.
$maxv_{rep}$	Maximum value of a reported data.
$minv_{rep}$	Minimum value of a reported data.
$max\sigma_{org}$	Maximum value of a standard deviation.
$min\sigma_{org}$	Minimum value of a standard deviation.
$b_{v}$	Scale factor of a Laplace noise with regard to the sensing value.
$b_{\sigma }$	Scale factor of a Laplace noise with regard to the standard deviation.
${†}_{i}$	Number of participants whose reported values were categorized into the *i*th bin.
${‡}_{i}$	Estimated number of participants whose true values were categorized into the *i*th bin.
**†**	$\left\{{†}_{1},\ldots ,{†}_{b_{n}}\right\}$.
**‡**	$\left\{{‡}_{1},\ldots ,{‡}_{b_{n}}\right\}$.

**Table 3:** Architecture of a deep neural network used in the experiment.

Layer ID	Description of Each Layer
1	Input Layer
2	Convolutional Layer
3	Convolutional Layer
4	Max Pooling Layer
5	Convolutional Layer
6	Convolutional Layer
7	Max Pooling Layer
8	Convolutional Layer
9	Convolutional Layer
10	Convolutional Layer
11	Max Pooling Layer
12	Convolutional Layer
13	Convolutional Layer
14	Convolutional Layer
15	Max Pooling Layer
16	Convolutional Layer
17	Convolutional Layer
18	Convolutional Layer
19	Max Pooling Layer
20	Convolutional Layer
21	Convolutional Layer
22	Fully Connected Layer
23	Output Layer
